# Coxsackie B Virus-Induced Cardiac Tamponade and Adrenal Insufficiency

**DOI:** 10.7759/cureus.45272

**Published:** 2023-09-14

**Authors:** Su Hyun Jeong, Fernando J Fuentes

**Affiliations:** 1 Internal Medicine, University of Nevada, Reno School of Medicine, Reno, USA; 2 Family Medicine, Renown Regional Medical Center, Reno, USA; 3 Internal Medicine/Pulmonary and Critical Care, University of Nevada, Reno School of Medicine, Reno, USA; 4 Pulmonology, Renown Health, Reno, USA

**Keywords:** pericardial effusion, adult internal medicine, adrenal insufficiency, cardiac tamponade, coxsackie b virus

## Abstract

We report a case involving a young male patient without a significant medical history who exhibited symptoms of fatigue, shortness of breath, chest and back pain, and syncope with vomiting. He was found to have adrenal insufficiency and cardiac tamponade requiring pericardiocentesis. Further inpatient workup revealed the patient had positive IgM and IgG antibody titers for the coxsackie B virus, which we believe caused his presentation. The coxsackie B virus strain can cause mild gastrointestinal to more severe cardiac and neurological complications, including meningitis and myocarditis. On rare occasions, the virus can appear in an unexpected fashion, such as in cardiac tamponade or hormonal disruption. This case raises attention to the broad manifestations of the virus and recognizing its more uncommon presentations.

## Introduction

The coxsackievirus is a form of *Enterovirus* in the Picornaviridae family that is typically spread through the fecal-oral route. Within the virus, there are 29 recognized serotypes belonging to either group A or group B [1]. With the number of serotypes that exist, it is unsurprising that the coxsackievirus manifests in many different fashions. The disease typically affects children, and both groups can cause fevers, rashes, upper respiratory symptoms, and aseptic meningitis. However, the illness is relatively benign, with 90% of infections being asymptomatic or causing a mild fever [2]. The coxsackie A virus group specifically is a common cause of hand, foot, and mouth syndrome. On the other hand, the coxsackie B virus can target the heart, causing symptoms of chest pain or palpitations. These symptoms are oftentimes managed supportively or with non-steroidal anti-inflammatory drugs but on occasion can progress to conditions including pericarditis, myocarditis, and heart failure. Less frequently, we see virus-induced cardiac presentations including cardiac tamponade. Pathophysiology for this presentation is poorly understood, and the virus is normally not on the differential.

Strains of the coxsackie B virus have been observed to target the pancreas, resulting in conditions such as acute pancreatitis or type 1 diabetes mellitus [3]. Other conditions involving the endocrine system are uncommon. Rarer still, few reports depict the virus simultaneously infecting the cardiac and endocrine systems.

Our patient showcases a unique scenario of the coxsackie B virus causing both cardiac tamponade and adrenal insufficiency. This case highlights the importance of awareness in identifying the virus and the need for further research regarding pathophysiology and treatment.

## Case presentation

This 30-year-old male without significant medical history arrived with complaints of chest pain and shortness of breath for the past three days. He was sitting up as he could not tolerate being supine. Just prior to arrival, his spouse noted a syncopal episode with some non-bloody, non-bilious vomiting.

**Figure 1:**
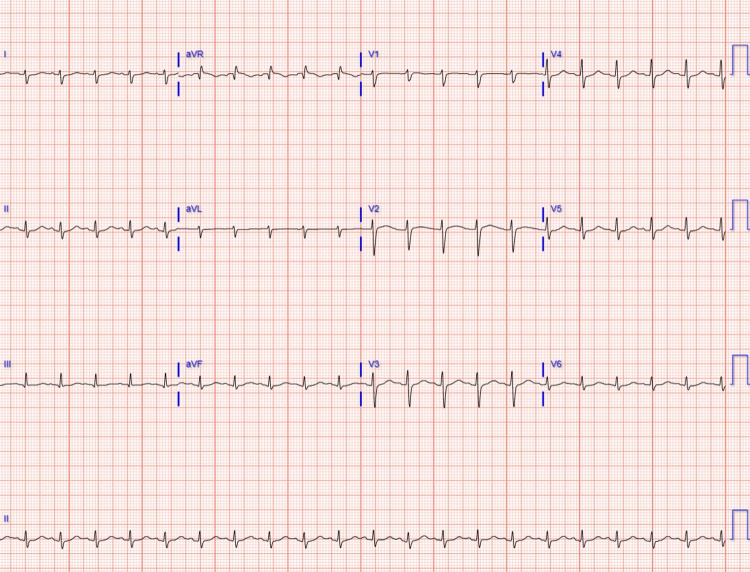
EKG on admission The patient’s EKG showed sinus tachycardia, left posterior fascicular block, borderline low voltage, extremity leads, and prolonged QT interval. EKG: electrocardiogram

Vitals on admission include a heart rate of 122 beats per minute, respiratory rate of 27 beats per minute, blood pressure of 63/48 mmHg, SpO2 of 90% requiring 4 L of oxygen, and a temperature of 96.8°F. On physical examination, the patient was alert and oriented ×3 with no focal deficits but was noted to be very lethargic. His heart sounds displayed a regular rhythm but were tachycardic and diminished. The patient was tachypneic on lung auscultation but had normal breath sounds. His chest X-ray demonstrated no acute cardiopulmonary abnormalities, and his electrocardiogram (EKG) showed sinus tachycardia, left posterior fascicular block, borderline low voltage (Figure 1), and a QTc of 522. Initial laboratory results are listed in Table 1.

**Table 1:** Initial chemistry panel on admission highlighting hypoglycemia, acute kidney injury, and metabolic acidosis versus chemistry panel on discharge four days later BUN: blood urea nitrogen, ACTH: adrenocorticotropic hormone

	Day of admission	Day of discharge	Reference ranges
Sodium	139 mmol/L	139 mmol/L	135-145 mmol/L
Potassium	3.3 mmol/L	4.3 mmol/L	3.6-5.5 mmol/L
Chloride	102 mmol/L	110 mmol/L	96-112 mmol/L
CO2	14 mmol/L	19 mmol/L	20-33 mmol/L
Glucose	62 mg/dL	125 mg/dL	65-99 mg/dL
BUN	26 mg/dL	29 mg/dL	8-22 mg/dL
Creatinine	3.35 mg/dL	0.99 mg/dL	0.50-1.40 mg/dL
AM cortisol	<0.2 ug/dL	0.7 ug/dL	0-23 ug/dL
C-reactive protein	5.69 mg/dL	-	0-0.75 mg/dL
ACTH	1,461 pg/mL	-	7.2-63.3 pg/mL
Troponin T	71 ng/L	-	6-19 ng/L

**Figure 2:**
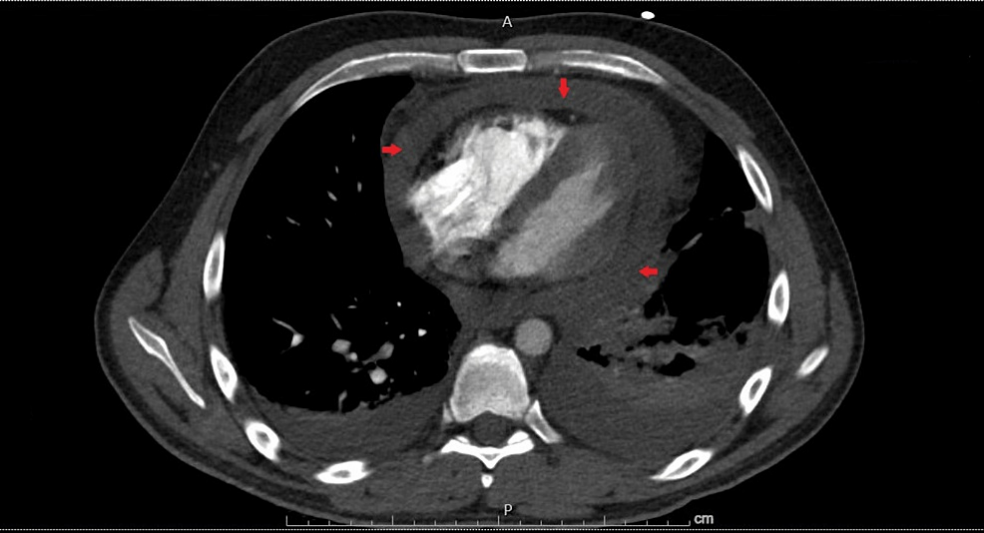
CT angiogram showing pericardial effusion (arrows) CT: computed tomography

Bedside focused assessment with sonography for trauma (FAST) revealed a large pericardial effusion with tamponade. Given hypotension and hypoglycemia, he was provided dextrose with fluid boluses and started on a norepinephrine drip. Cardiology was consulted, and computed tomography (CT) angiogram ruled out aortic dissection but again showed pericardial effusions and concerns for tamponade (Figure 2).

Once pressures improved, the patient went to the catheter laboratory for pericardiocentesis where 450 cc of fluid was removed (Table 2).

**Table 2:** Pericardial fluid studies WBC: white blood cells, RBC: red blood cells

	Value
Body fluid color	Yellow
Total WBC	6,700 cells/uL
Total RBC	<2,000 cells/uL
Polys	93%
Lymphs	3%
Mononuclear cells	12%
Fl mono-macrophages	4%
Total protein	5.1 g/dL
Albumin	3 g/dL
Culture	Negative

Post-procedure, vitals quickly stabilized. Blood work revealed the patient had 1:320 positive antibody titers for the coxsackie B virus and had adrenal insufficiency with low cortisol levels. Treatment with colchicine and steroids was initiated, and 48 hours later, transthoracic echocardiogram was negative for pericardial effusion. He was discharged the following day with recommendations for follow-up with endocrinology and his primary care physician.

## Discussion

The coxsackie B virus traditionally affects adults with a benign and uneventful course. This case functions as a demonstration that the virus can sometimes present in a unique fashion. The coxsackie B virus knowingly causes pericarditis, but the pathophysiology for tamponade is unclear with only a handful of reported cases. One case involving a 58-year-old male with hemorrhagic pericardial effusions and tamponade from the coxsackie B virus postulated that since the virus has been shown to cause chronic infection, the long-term damage to the myocardium could in theory result in fluid buildup [4]. There are multiple explanations that focus on the virus’ ability to cause prolonged infection. Some research uncovered a process of antibody-dependent enhancement where antibodies paradoxically increase the ability for infection by failing to neutralize the virus while still bringing it closer to its target cell [5]. This in turn would assist the virus in invading cells, lengthen the duration of infectivity, and finally elicit a stronger inflammatory response. Another study detailing the chronicity of the coxsackie B virus found that the virus may have a latency state where it slowly replicates within quiescent G(0) cells [6]. When the same study observed these dormant cells progress through the cell cycle into the G1 and S phases, the cells began to rapidly increase viral load production. A third paper shed light on the presence of residual RNA long after acute symptom resolution that may be toxic to cells and contribute to a prolonged inflammatory response [7].

However, what sets this report apart is the additional component of virus-mediated adrenal insufficiency. There are examples of the coxsackie virus affecting different aspects of the endocrine system. Acute pancreatitis is a well-known complication seen from coxsackie virus infections [8]. Unfortunately, the literature is sparse when it comes to understanding the effects of the virus on the adrenal glands. What is known from previous studies is that the coxsackie B virus has been reported to accumulate in the adrenal cortices [9]. Given the virus’ ability to persist in its host, it is reasonable to assume direct cellular injury through increased inflammatory factors in the same manner as with cardiac tissue. A few cases in children even observe adrenal necrosis as a result of direct cellular injury [10].

A second process by which the virus causes adrenal disruption is through transient suppression of the hypothalamic-pituitary system. There are instances of temporary hypogonadism seen in coxsackie virus-mediated encephalitis that resolves shortly after the resolution of infectious symptoms [11]. Although our patient had neither neurological symptoms nor sex hormone deficiencies, the intimate relationship between the hypothalamic-pituitary system and adrenal glands suggests a connection. We acknowledge that the evidence here is limited as these other cases showed a remarkably isolated disruption of gonadal hormones while maintaining adequate levels of thyroid or adrenal function. Further research would be needed to explore this relationship.

## Conclusions

The coxsackie virus clearly manifests in various and hard-to-predict ways. Most of the presentations are benign and ultimately harmless. However, we cannot be complacent when confronted with the disease. The virus on occasion can be challenging and sometimes fatal for the patient, such as in cardiac tamponade or adrenal insufficiency. The pathophysiology of these conditions and the coxsackie B virus is unclear and not extensively researched. This case brings awareness to better understand the virus when dealing with these uncommon findings.
